# Hong Kong’s new normal: Remaking authorized discourses of “special administration,” 2017–2022

**DOI:** 10.1177/23996544241227159

**Published:** 2024-01-20

**Authors:** Jamie Peck, Chris Meulbroek, Dimitar Anguelov

**Affiliations:** 8166 UBC, Canada

**Keywords:** Governance, Carrie Lam, discourse, Hong Kong, China

## Abstract

The paper presents a critical discourse analysis of the five annual policy addresses of Hong Kong’s chief executive, Carrie Lam Cheng Yuet-Ngor, whose term of office (2017–2022) spanned the most tumultuous episode in the modern history of the territory, one bisected by an extended season of street protests and the subsequent imposition of a national security law. Read as an authorized, real-time transcript of governmental rationalizations and responses, delivered in the first-person voice of Hong Kong’s most senior politician, the annual policy addresses narrate a crisis-mediated shift from a postcolonial redoubt of liberal capitalism to an outpost of China’s national-security state. Although the “one country, two systems” formula enshrined in Hong Kong’s Basic Law retains its official status, a distinct transformation is evident, from the evocation of “two systems” as a wedge against one-country assimilation, to an apparently new normal based on the principles of securitized, “one-country” integration. In the process, the chief executive increasingly embraced the received discourses and sanctioned priorities of the Chinese Communist Party in her depictions of a “New Paradigm” for the governance of Hong Kong, founded on the “steer of the Central Government” with the “cooperation” of the government of the Special Administrative Region.

## Introduction: Special administration

Hong Kong has long been subject to various forms of “special administration.” The Sino-British Joint Agreement, enshrined in Hong Kong’s Basic Law, pledged that the former Crown colony would retain its “capitalist system and way of life … unchanged” for 50 years following the resumption of Chinese sovereignty in 1997. The resulting “one country, two systems” (1C2S) arrangement conferred a “high degree of autonomy” to the Special Administrative Region of Hong Kong (HKSAR), along with a sense of suspended animation—implying a future postponed, along with an inscrutable expiry date. Deng Xiaoping saw Hong Kong as the lodestar for a modernizing China, during what would be a transformative half century to 2047, with the People’s Republic “approach[ing] the economic level of advanced countries by the end of that time” (quoted in Au, 2020: 6). At the halfway stage of this constitutional timetable, a different kind of future beckons. Following a quarter century of depressed growth, accelerating inequality, and weak leadership, Hong Kong drew global attention in 2019-2020 for an extended and escalating season of protests, which were brought sharply to an end by the imposition of a far-reaching national security law (NSL). With prominent democratic activists in prison or exile, and independent local media outlets either closed down or cowed,^1^ Hong Kong’s postcolonial experiment in “semi-democracy” has come to an end, at least for now (Fong, 2021; Hui, 2020; Lee, 2022). Refusing to countenance any compromise of its “comprehensive jurisdiction” over the territory, the central authorities in Beijing continue to declare “unswerving” commitment to the 1C2S principle of relative autonomy, the meaning of which has been redefined and “clarified” by the party-state (Cross, 2022; Xi, 2022).

Carrie Lam Cheng Yuet-Ngor served as the chief executive of the HKSAR from 2017 to 2022, spanning the most tumultuous episode in the modern history of the territory. She would confess at times to being “deeply worried about the future of Hong Kong,” but following the passage of the NSL asserted that the city was ready to “start over again” with a “new blueprint” that reflected the “steer of the Central Government” and the “cooperation” of the HKSAR (Lam, 2020: 80, 2021: 79; Xi, 2022). The received discourses, approved phraseology, and sanctioned priorities of the Communist Party of China (CPC) have since become increasingly pervasive, Lam subsequently becoming “increasingly formal in her interactions, her conversation and pronouncements carefully morriring the language favoured by Beijing” (MacSweeney, 2023: 341; R Chan, 2023b). And yet even as the letter of the Basic Law remained unchanged, the *meaning* of 1C2S had evidently shifted, as Lam explained in what would be the last of her annual policy addresses:

So long as the HKSAR firmly observes and upholds the principle of “One Country” and fulfils the requirement of “patriots administering Hong Kong,” the “Two Systems” will definitely thrive and flourish, and Hong Kong will continue to be cherished by our country (Lam, 2021: 80, emphasis added).

Despite her intention to seek a second term as Hong Kong’s chief executive, Lam failed to receive Beijing’s backing, stepping aside to allow John Lee, the HKSAR’s former security chief, to run as her successor. Observers have taken Lee’s de facto appointment as chief executive in 2022, “from a party shortlist of one,” to be a marker of Hong Kong’s transition from a liberal to a securitized mode of governance, the city’s policing budget having surged by 45% in the preceding five years (Economist, 2022: 44; see also Vukovich, 2022).

This paper investigates how Hong Kong’s abrupt transformation—from a (post)colonial redoubt of liberal capitalism to an outpost of China’s national security state—has been accommodated and narrated in high-level governmental discourse, taking as its focus the five annual policy addresses delivered during Lam’s term of office, the prominent status of which has been equated with state-of-the-union speeches. Critical histories of this period are already beginning to weigh the implications of an apparent phase-shift in political culture, geopolitical relations, and democratic development (see Au, 2020; Cheung, 2022; Dapiran, 2020; Hung, 2022; Lee, 2022; Lim, 2022; Vukovich, 2022). Informed by the methodology of critical discourse analysis, with its signature concern to analyze the exercise of power and the mediation of “macropolitical” movements through discourse (Fairclough and Fairclough, 2012; Flowerdew, 1999, 2012), the present paper seeks to make a distinctive contribution by documenting movements in the voice and messaging of Hong Kong’s senior administrator at the “microlinguistic” scale, explored within the strict parameters of a continuous corpus of high-level, authorized discourse, recognized for its meticulous scripting, strategic significance, and public visibility. Using this “controlled” and disciplined sampling method, rather than engaging source materials more selectively, the paper traces and interprets lines of dis/continuity in this preeminent transcript of governmental rationalizations, representations, and reactions, documenting the discursive “signals” of Hong Kong’s transition to a new normal. In the first-person voice of the territory’s most senior politician, this manifests as a crisis-mediated shift from an inherited style of liberal paternalism, predicated on an incumbent, “two-systems” normativity, to a mode of authoritarian control with Chinese characteristics, anticipating a future governed under the sign of “one-country” integration. The paper documents how this transformation has been realized through the purposeful (re)narration of real-time events and circumstances, with implications for the subjects, objects, and spaces of governmental regulation. As such, the paper can be read as a situated commentary on the vicissitudes of late-neoliberal governance, in this case centered on what was once a quintessential truth spot for the free-market project since in the throes of a transition toward a “hard shell” model of securitized government (cf. Peck and Theodore, 2019).

Beginning with a sketch of Carrie Lam’s Hong Kong, the paper then moves to a close textual analysis of the chief executive’s five policy addresses. This is predicated on inductive coding and (ac)counting, engaging three cross-cutting themes identified in this evolving, real-time transcript: first, “rebooting governance” traces the shift from Hong Kong’s classical model of small-state pragmatism through to the authoritarian reinterpretation (or “correction”) of the 1C2S formula after the NSL; second, “relocating economy” documents an evolution from the light-touch regulation of the pre-crisis period to the redoubled imperative to assimilate with China’s pattern of development; and finally, “reordering the social” examines the transition from the incumbent stance of paternalism and presumed consent to the new order of securitized control and moral leadership. It concludes by returning to the question of Hong Kong’s “special administration,” characterizing Lam’s term of office as a punctuated transition between governmental orders. Her administration began on the familiar (if ambiguous) terrain of postcolonial governance, where the 1C2S formula preserved Hong Kong’s concessionary state of exception. It would end with a quite radical recalibration of this formula: once interpreted as a wedge against one-country assimilation (see Wesley-Smith, 1996; cf. Lui, 2020; Perlez, 2020), 1C2S has come to function as a “bridge,” or lever, in an accelerating but uncertain process of integration, subject to the primacy of one-country rule.

## Carrie Lam’s Hong Kong, 2017–2022

Hong Kong’s governance system concentrates (local) power in the hands of the chief executive, echoing the role previously performed by colonial governors. Authority and relative autonomy are not intrinsic to the SAR itself but are instead “granted” by the party-state in Beijing (Summers, 2021: 42; Ma, 2018). There is no formal separation of powers, with local elections playing a circumscribed role, more so as a (once elevated) degree of regional autonomy has given way to a supervised form of “semi-autonomy,” subject more expressly than before to the Chinese constitution and the strategic interests of the CPC (Fong, 2018; Ma, 2018; Vukovich, 2022). Interpretations of these transformations are unavoidably political and often polarizing. Ching Kwan Lee (2022: 12) has argued that, “Beijing’s political design for Hong Kong was to make the system accountable to itself rather than to the Hong Kong people,” the Basic Law having been “imposed as the mini-constitution of the SAR, already prepar[ing] the way for Beijing’s intervention.” For Ngok Ma (2018: 40), “systemic voids” in the Basic Law have fostered not only ambiguity but governmental dysfunction, due to the “fragmentation of executive authority and state capacity,” which Brian C. H. Fong (2018: 65–66) identifies as the root cause of inbuilt vulnerabilities, including legitimacy deficits and crises of governance (see also Lui, 2020).

Since 1997, a succession of generally unpopular chief executives has assumed the task of administering the HKSAR, with Carrie Lam achieving the distinction of being the least popular to occupy the role (McLaughlin, 2020; Vines, 2021). A career bureaucrat, as a candidate for office in 2017 Lam was known to be the preferred choice of Beijing. Media assessments of the time portrayed her as a “conciliatory figure,” distrusted by pro-democracy activists but supported by establishment interests; her reputation was that of a “fiercely competent administrator,” rather than a sophisticated politician. It has been said that Lam’s career was defined by loyalty, initially to the colonial service, pre-1997, and then “even more [so] to the Mainland Communist regime” (Khan, 2022: A8; Wang, 2021: 1; Vines, 2021: 317; MacSweeney, 2023: 337–338).

Lam assumed office in 2017 pledging to “heal the divide,” an implicit acknowledgement that Hong Kong politics had splintered around the fraught questions of democratization and relations with Beijing (McLaughlin, 2020). The two decades since 1997 had been defined by Beijing’s “tightening grip,” marked by repeated attempts by the party-state to extend national security legislation and to exercise veto power over local elections (Cheung, 2018). Large-scale protests and social movements against these measures exacerbated already deep-seated divisions between a pro-autonomy majority and pro-integration minority (Cartledge, 2017; Eagleton, 2020; Lee, 2022; Lui, 2020; Vukovich, 2022). This further intensified the dilemma confronted by every one of Hong Kong’s chief executives, that of “serving two masters,” the people of Hong Kong and the party-state in Zhongnanhai (Lam, 2020: 3; Ma, 2018; MacSweeney, 2023). If Lam’s candidacy was itself a product of these challenging circumstances, the events of her term of office would reaffirm them beyond doubt.

Predictably, it was a national-security issue—the Lam administration’s proposal to allow extradition to mainland Chinese courts—that triggered the first of the large-scale street protests, in June 2019. Although the numbers remain contested, around one million citizens joined peaceful marches to protest the proposed legislation, to which the local authorities responded first with intransigence and subsequently, as tensions rose, with combative policing. Beijing’s decision to impose the NSL, following the protracted failure of Lam’s government to defuse the crisis, came as prelude to a comprehensive crackdown on political dissent and the independent media. This brought an abrupt end to a year of civic protest, counter-mobilization, and unrest, raising searching questions about the governance (if not fate) of Hong Kong and its long-cherished cultural freedoms. Having ridden out the storm, Lam’s final 2 years in office were overshadowed by the COVID-19 pandemic, the territory’s increasingly repressive “order” being marked by a palpable political chill and deepening legitimacy deficits. In the process, the hand of Beijing in Hong Kong—in part through the work of the innocently named Liaison Office—has significantly strengthened (see Cheng and Cheung, 2022; Lui and Fong, 2018). The voice of Beijing, too, would increasingly echo through the channels of an official discourse that prior to 2019 had been barely permeated by partyspeak (Maizland, 2022; R Chan, 2023a).

### Addressing policy

Since their introduction in the early 1970s, the policy addresses of Hong Hong’s chief executive have established the framework, terms, and tone for the governance of the territory. The most scrupulously planned and scripted events in the political calendar, the annual addresses represent the most rarefied of governmental texts. As set-piece public statements of the administration’s strategic outlook, policy priorities, implementation plans, diagnoses and rationalizations, they possess the uniquely authoritative status of the considered word and practice of executive-led government. This said, there remains a sense in which the addresses must also cultivate consent, seeking to secure legitimacy for governmental actions undertaken or proposed, albeit in a context effectively insulated from democratic input.

To target these high-level governmental texts as (re)sources for CDA is to prioritize just one axis of this broad-ranging and cross-disciplinary methodological approach, which in other applications might be deployed to probe the “reception,” reciprocation, and societal effects of (dominant) discourses; the (re)production of hegemony and common sense; discourses of opposition and resistance; and much more (see Weiss and Wodak, 2003). The following analysis prioritizes just one of the strategic objectives of this approach: exploring the *articulation* between texts and (sociopolitical) contexts, or as this is sometimes expressed, between the micro and the macro. This is not to assume a straight-line relationship between specific texts and the configuration of political power, less still between the policy pronouncements of politicians and the eventual outcomes of governmental interventions and political struggles. CDA has in fact been vulnerable to critique on the grounds of an observed “tendency to jump too quickly to the macro context,” from a theoretically informed and selective reading of chosen texts to wide-angle conclusions pertaining to movements in politics, power, societal norms, and ideology, with textual evidence marshalled to confirm the theoretical and political presuppositions of analysts (Breeze, 2011: 513). Sympathetic critics argue that, in practice, the methodological procedures and interpretative routines of CDA are inadequately codified and insufficiently systematic, resulting in tendencies to instrumentalize social-theoretical precepts through the selective sampling of texts, thereby truncating and foreclosing more inductive and exploratory moments of empirical analysis (Breeze, 2011; Verschueren, 2011).

The present study cannot claim immunity from such critiques, although they have informed a more disciplined and procedurally inductive methodological strategy, predicated on a multi-stage, systematic analysis of a whole-text sequential corpus of governmental speech-acts, linked to shifting sociopolitical circumstances as represented within the texts themselves. Evidently, this exclusive focus on governmental texts cannot address issues of reception and resistance, or the wider terrain of political discourse in Hong Kong.^2^ On the other hand, where there is utility in this knowingly one-dimensional mode of inquiry, which takes as pregiven the content and boundaries of the analysis (including text selection itself), it is that critical attention is methodically focused on connections and movements within an *authorized sequence of texts*, as determined and documented by the recognition of patterns, shifts, and continuities. This tends to foreground real-time changes in governing discourses and authorized narratives, those that combine official interpretations of contemporaneous events, policy challenges, imperatives and opportunities with attempts to rationalize, justify, and legitimize an array of appropriate and necessary responses.

This is not to stake a claim to inductive neutrality or descriptive objectivity, but it is to open up the questions of how Hong Kong’s governing authorities navigated and narrated what was both a crisis registering on the scale of global spectacle, and a crisis-driven shift in the very character of governmental philosophy and practice. For all the official claims to governmental continuity and integrity, and to the uninterrupted sanctity of 1C2S provisions, Lam’s post-crisis “new blueprint” differs sharply from the previous blueprint (Lam, 2020: 80, 2021: 79), just as Xi’s “new stage” differs from the previous stage (Xi, 2022: 10). Analytically, there is no need to posit (or superimpose upon the textual record) an imagined transition model—as if from an ideal-typical state of liberal-capitalist freedoms to some absolute form of authoritarian-statist control—since the objective of the present analysis is instead to calibrate, document, and illustrate what has been an incomplete process of qualitative transformation, as narrated by those occupying the highest offices of territorial government.

The resulting span of the analysis, coinciding with Lam’s five-year term of office, amounts to a relatively compressed historical arc objectively marked by a dramatic realignment of political and cultural norms. This has pivoted around a disruptive inflection point, the moment of protest and securitization during 2019–2020, which has been followed by declarations of a “new stage” and the restoration (or reconstruction) of governing order in the context of a “new paradigm for a new future” (Xi, 2022: 10; Lam, 2021: 13). In accordance with the previous discussion, rather than asserting (or imposing) an a priori reading of this period, the methodological strategy adopted here leans toward the empirical and the inductive: to read, code, and analyze the texts themselves for indications and representations of the shifting sociopolitical terrain, and the articulation (in all senses of the word) of responses to these circumstances. The paper seeks to detect—and then depict—the “signal” of these objectively turbulent circumstances in authorized narratives. Systematizing wherever possible—through frequency counts, content coding, the mapping of keywords like freedom, security, order, and so forth—the methodological strategy began with three independently-conducted rounds of “open” coding, reading for content and context, subsequently refined according to an emergent cluster of recurring themes, concerning modalities of governance, economic statecraft, and social regulation.

**Figure 1.**
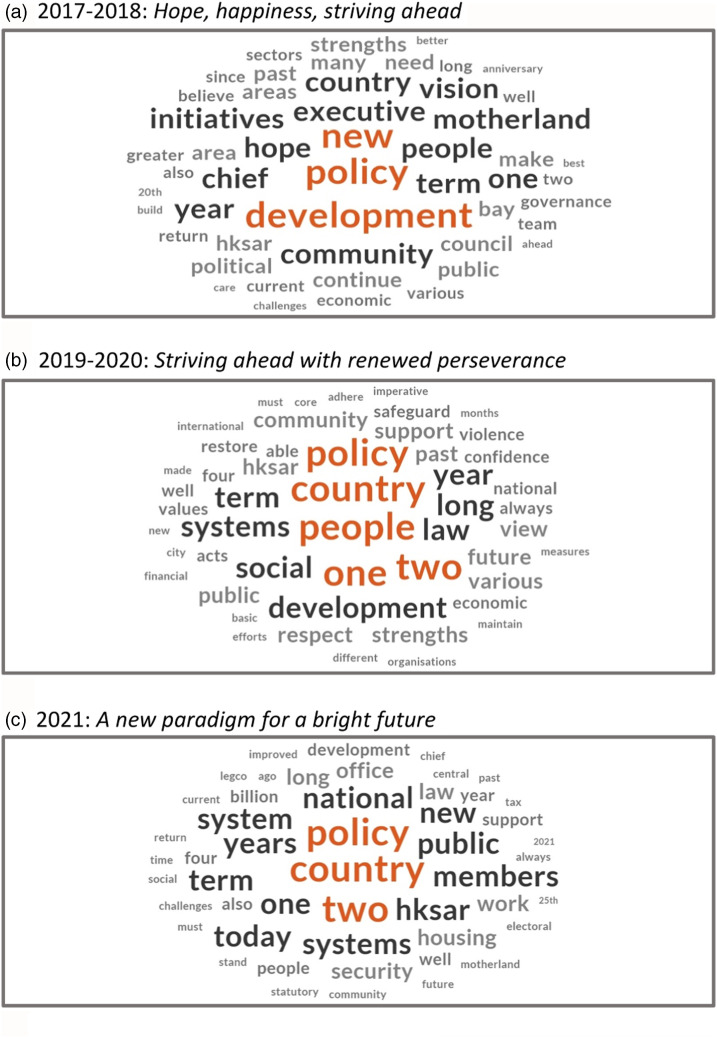
Word clouds of the forewords and concluding statements of Hong Kong annual policy addresses, 2017-2018, 2019-2020 and 2021.

These analyses of twists and turns of Hong Kong’s official discourse through the span of the Lam administration reveal certain continuities—in postcolonial administration and detached, managerialist governance—punctuated by shifts and realignments that are at times abrupt but in many cases also cumulative. The word clouds at Figure 1 and the contents pages summarized at Table 1, which precis the policy addresses that bookended Lam’s administration and the period of crisis and restoration in between, telegraph a storyline of development interrupted. The chief executive’s inaugural address of 2017 promised “hope and happiness,” braiding together, in terms not dissimilar to those of her predecessors, a growth-oriented and developmentalist mission with an optimistic, can-do outlook, recycling staple themes and generic goals concerning good governance, economic diversification, and liveability. In contrast, the truncated 2019 address, delivered in the throes of the crisis, evoked the image of a shared home violated, while the 2020 address, following on the heels of the NSL, pledged to persevere in the face of “interference by external forces” and the “radical acts of rioters.” Lam’s first two addresses led with first-person declarations of the chief executive’s governing philosophy and vision, each chapter being prefaced by passages entitled “My Belief.” This formulation disappears in 2019 text, never to return. Instead, the dominant motifs after 2019 are systemic: 1C2S being the most prominent, but also law, statutory, security, safeguard. Lam’s final address deferred explicitly to the “steadfast” principles of 1C2S, courting public support for a “new paradigm” of territorial governance, “building together,” in the service of national unity and development.

**Table 1.** Shifting contents: Policy addresses of the Hong Kong chief executive, 2017–2021.

2017: We connect for hope and happiness	2018: Striving ahead rekindling hope	2019: Treasure Hong Kong: Our home	2020: Striving ahead with renewed perseverance	2021: Building a bright future together
[35,000 words]	[40,700 words]	[7700 words]	[25,200 words]	[25,300 words]
I. Introduction: A new beginning	I. Foreword: Striving ahead	I. Foreword	I. Foreword: Striving ahead	I. Foreword: A new era
**II. Good governance**	**II. Good governance**	**II. Housing**	**II. Full support of the central government**	**II. Steadfastly and successfully implementing “one country, Two systems”**
**III. Diversified economy**	III. Housing and land supply	III. Land supply	**III. Upholding “one country, Two systems”**	**III. New paradigm for a new Future**
IV. Nurturing Talent	**IV. Diversified economy**	IV. Improving People’s livelihood	IV. Navigating through the epidemic	IV. New impetus to the economy: Integration into the national development
V. Improving people’s livelihood	V. Nurturing talent	**V. Economic development**	V. New impetus to the economy	V. Increasing housing and land supply
**VI. Liveable city**	VI. Improving people’s livelihood	VI. Closing Remarks	VI. Increasing housing and land	VI. Building a liveable city
VII. Connecting with young people	**VII. Liveable city**		VII. Building a liveable city	VII. Continuously improving People’s livelihood
VIII. Conclusion	VIII. Connecting with young people		VIII. Continuously improving People’s livelihood	VIII. Nurturing Talents and youth development
	IX. Conclusion: rekindling hope		IX. Closing remarks: Renewed Perseverance	IX. Emerging from the epidemic
				X. Closing remarks: United for a Future

				2021 supplement: Report on Hong Kong’s business environment
				[62,000 words]
				1. Black-clad violence and the US’ strategy of suppressing China …
				2. The formulation and implementation of the Hong Kong national security law and the principle of “patriots administering Hong Kong” …
				3. The new Hong Kong back on track

Note: Emboldened text indicates continuation or change in governing keywords

If the first act of Lam’s administration occupied familiar terrain, reiterating Hong Kong’s signature brand of free-market rhetoric and small-state paternalism, the new normal anticipated in its second act spoke to a radically altered course (and cause). The space once reserved for pragmatic, market-friendly governance has been progressively occupied by an assertive party-state, the uncompromising voice and tentacular powers of which have been likened to those of an “organizational emperor” (Zheng, 2010). Of course, these powers are exercised by proxy and “at a distance” in the case of Hong Kong, the still-formally-separate “system” of which is permitted a higher degree of autonomy than any other jurisdiction in the country. But the voice of Beijing is more audible, its hand more visible. China’s party-state was operationally and literally absent from the first three of Lam’s policy addresses (2017–2019), which ran to some 231 cumulative pages (and 83,000 words of text), save for a single, innocuous reference to a line from one of President Xi’s speeches, a trite aphorism about youth as the bearers of a “bright future” (Xi, quoted in Lam, 2018: 91). In Hong Kong’s new normal, post-2019, the customary lexicon and governing ethos of the party-state are not only more evident, they have become objects of performative deference. For example, the two policy addresses delivered since the passage of the NSL frame the “smooth return of Hong Kong to the Motherland [as] an integral part of the CPC’s century-old cause,” expressly recognizing the overriding authority of the National People’s Congress, not only as “the highest organ of state power,” but also as the ultimate arbiter of the “correct” interpretation of 1C2S and other such “issues left over by history” (Lam, 2021: 4; Lam, 2020: 7). The 2020 address expressly confirms the overall jurisdiction of the CPC, with its overarching responsibility for “safeguard[ing] national sovereignty, security and development interests,” while Lam’s final address, in 2021, pledged to “stay true” to the original intent of 1C2S in “letter and spirit,” as decreed by the Fourth Plenary Session of the 19^th^ Central Committee of the CPC (Lam, 2020: 22, 80, 2021: 4).

Taken together, Lam’s policy addresses narrate a dogleg journey from the pronouncements of a cosmopolitan and extrospective HKSAR, projecting its own voice, confident of its place in the world, and exercising a modestly proactive mandate in the received language of liberal governance, to a much more restrained and restrictive voice of a subordinate city-region, striving to conform and comply with *national* priorities in the domains of security, social order, integrated development, and geopolitics. This pivotal realignment, which has been repeatedly affirmed under the leadership of John Lee,^3^ measurably defined the second act of Lam’s five-year term. The contents pages of her five addresses reproduced at Table 1 attest to what is now described, in the official discourse of both Hong Kong and Beijing, as an altered “paradigm.”

Lam’s two pre-crisis policy addresses, both of which led with titular references to “hope,” occupied the customary terrain of mainstream metropolitan governance, routinely addressing a standard-issue list of public-policy domains: efficient administration, economic diversification, and quality of life. The tenor is businesslike, idealistic, and inclusive. Yet even the pre-crisis Carrie Lam had her red lines. Closing the door to democratic development, she pointedly declared in her maiden address that “we cannot ignore the reality and rashly embark on political reform once again,” a phrase that is repeated, verbatim, in 2018 (Lam, 2017: 9; Lam, 2018: 11). Commitments to political containment, social order, and the slow-walking of electoral reform (both legacies of the suppression of the 2014 Umbrella Movement) were integral to Lam’s political identity from the outset.^4^ The 2018 address baldly stated her (government’s) position, “the HKSAR Government and I will not tolerate any acts that advocate Hong Kong’s independence and threatens (sic) the country’s sovereignty, security and development interests” (Lam, 2018: 3). This vision combined the embrace of an open economy with stunted political development at home, safely within governing parameters (pre)authorized by Beijing.

Against this backdrop, the effects of the year of “chaos and fear,” 2019-2020, are plainly evident (Lam, 2019: 1). Lam’s mid-crisis address took the form of a terse and “focused” statement that was just a fraction of the usual length. It expressed a desire to return to bread-and-butter issues like housing, livelihood, and economic development, while disregarding the programmatic demands of the protest movement, which included calls for Lam’s resignation (Lam, 2019: 4, 1; Tsang et al., 2019). Delivered in a Legislative Council (Legco) chamber ringed by security personnel, the speech was interrupted by pan-democratic lawmakers chanting the protest slogan, “five demands, not one less,” which was also projected onto the wall behind the speaker’s podium.^5^ Lam was forced to abandon the presentation, later replaced by a pre-recorded video. The address itself was not well received. The *South China Morning Post* reported that it had been “universally panned,” particularly on the grounds that it offered “no political solutions to restore normalcy to a city rocked by more than 4 months of social unrest” (Tsang et al., 2019: 1). Beijing, on the other hand, evidently approved of Lam’s hold-the-line stance.^6^

**Figure 2.**
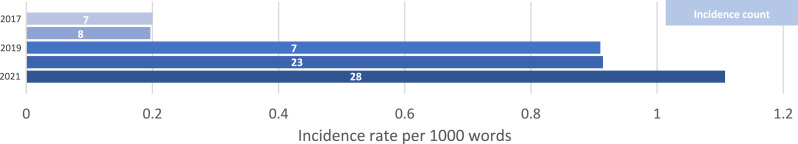
Governance keywords: “one country, two systems” in Hong Kong policy addresses, 2017-2021.

The policy addresses that Lam presented in her second-act (following the passage of the NSL, the imposition of restrictive electoral reforms leading to the unseating of opposition lawmakers, and the resignation of allies in protest) bore the hallmarks of post-democratic, one-party rule: “Unlike in the past,” the *Post* observed, “the atmosphere at Legco is [now] muted” (SCMP, 2021). Notable for their more formal, prolix, and constitutionally laden tone, the addresses of 2020 and 2021 feature new chapters devoted to (re)statements of the “Full Support of the Central Government,” to the HKSAR’s “steadfast” determination to “uphold” 1C2S, and to unilateral announcements of the advent, in familiar party-speak, of a “New Era” and “New Paradigm for a New Future” (Lam, 2020: 4, 9; Lam, 2021: 1, 15, 9). Prior to the crisis, 1C2S established taken-for-granted parameters for governmental discourse and action, observed mostly in “silence.” In contrast, since 2019 there has not only been a marked upturn in the “naming” of 1C2S, as recorded in frequency counts (see Figure 2), these elemental conditions of Basic Law have become more overbearing, their restatement being accompanied by instruction on the need for “clarification,” improved public understanding, and compliance.

Lam’s discursive style was initially characterized as a form of pro-establishment Confucianism, featuring appeals to consensus, pragmatism, and traditional values; disciplined and controlled, it tended to hew closely to authorized scripts and routines (Feng and Wu, 2015; Lim, 2018; Ng, 2020). Post-crisis, the chief executive’s voice became measurably more abrasive and uncompromising, “mirror[ing] that of the Chinese Communist Party, with its mix of strident denunciations and bureaucratic jargon” (Wang, 2021: 1; MacSweeney, 2023). She would speak of her steadfast resolve, intolerance of disorder, and resentment of “foreign interference,” interspersed with sycophantic declarations of gratitude to Beijing (Lam, 2020: 9, 12). For Lam, the imperative to “relaunch” Hong Kong, and to get the city “back on track,” weighed as a “heavy responsibility,” deeply and personally felt (Lam, 2020: 80, 1; Lam, 2021: 79). Her professed style of governance became notably more strident and determined, foreshadowing a mode of government with a much heavier hand.

This more combative stance appears in an extended supplement to the 2021 policy address, an innocuously titled *Report on Hong Kong’s Business Environment*. What would previously have been a business-like restatement of competitive advantages, commitments to market-friendly regulation, and coming opportunities, the 74-page report was instead largely given over to denunciations of rioting, “black-clad violence,” and the foreign policy of the United States (see Table 1), a malevolent combination of local and overseas threats. Only in its final chapter does the supplement turn to the concerns of mainstream economic development (digital capabilities, lifestyle amenities, robust financial governance, rule of law), before concluding that the

raging violent riots and the US’ unreasonable economic suppression of Hong Kong once dealt a severe blow to the economy and business environment of Hong Kong. Yet, following the implementation of the NSL … social stability and the business environment of Hong Kong have been quickly restored …The society has returned to rationality. Public order has been restored and riots are no longer seen (HKSAR, 2021: 67).

Here and elsewhere, “restoration” assumes the status of a dominant motif in post-crisis discourse, but designating a new normal rather than a simple “return.” Hong Kong now found itself in the crossfires of an increasingly acrimonious trade war between the United States and China, exacerbated by rising geopolitical tensions.^7^ Displacing the familiar imagery of the “gateway economy,” where East meets West and China connects with a liberalizing world, in increasingly Manichean terms it was now necessary to take (and defend) the Chinese side, while simultaneously attempting to placate and reassure international investors. The report declared that “Hong Kong’s financial market has remained stable” (accompanied, somewhat incongruously, by a statement from the American Chamber of Commerce in Hong Kong that the NSL “does not impact” commercial law), followed by a blunt reiteration of a Basic Law provision that would previously have been left unsaid: “the socialist system and policies will not be practiced in Hong Kong … [T]he HKSAR maintains its previous capitalist system” (HKSAR, 2021: 28, 40, 41).^8^

## Narrating Hong Kong’s new normal

Turning now to a thematically organized critical discourse analysis of Lam’s policy addresses, this section of the paper documents three transformations in the high-level discourse of the HKSAR: the first concerns the effective curbing of Hong Kong’s once “highly autonomous” status, along with a revisionist reading of 1C2S as a mechanism for coordinated development; the second focuses on the reorientation of economic-development discourse, from free-trading cosmopolitanism toward a supporting role in the project of national development; and the third traces the shift in social-policy discourse from a paternalist presumption of common values to an authoritarian preoccupation with corrective and “patriotic” education.

### Rebooting governance: from “autonomy” to coordinated development

Prior to the crisis of 2019, Lam was publicly advocating a cautious evolution of the governing posture of the HKSAR, adapting the classically liberal model that had long been the subject of celebration in official discourse (based on “free” markets, pro-corporate regulation, conservative budgeting, and fiscally “responsible” service provision) with a new accent on “facilitation” and proactive policymaking:

the Government should be visionary, scrutinise existing policies and measures pursuant to policy objectives, remove obstacles for our industries, and strengthen co-ordination and co-operation across government bureaux and departments (Lam, 2017: 3–4).

This marginally more proactive position was in turn predicated on the distinctive leverage (and “advantages”) provided by 1C2S compact, which in 2017 was named not only as a “unique asset,” but “the best institutional arrangement to ensure Hong Kong’s long-term prosperity and stability (Lam, 2017: 1). At the time, 1C2S and the “Hong Kong model” were typically invoked with liberal inflections (see Peck et al., 2023), as a source of (relative) autonomy amounting to a “freedom” to capitalize on Hong Kong’s advantageous position as an “open” international gateway economy and leading center for global finance.

Lam’s pre-crisis policy addresses received generally positive coverage in both Hong Kong media and in state-authorized outlets on the mainland. The editorial pages of the *South China Morning Post* praised her maiden address for its classically neoliberal emphasis on “higher growth and lower taxes,” supplemented by efforts to diversify the economic base (Siu and Yeung, 2017: 6; A Wu, 2017a: 11). Commentary in the *China Daily*, in effect the mouthpiece of the CPC, struck similar notes, welcoming Lam’s vision of a “Golden Era” of information-technology centered development with its emphasis on the “key issue of long-term competitiveness” (W Wu, 2017b: 5; Ho, 2017: 7). But if this represented the incumbent (rhetorical) style of economic governance, it was about to be disrupted. In the wake of the 2019 crisis and the NSL of 2020, Hong Kong’s timidly proactive voice in economic development was brusquely subordinated to the imperatives of *national* policy, with its emphases on “dual circulation” and macroregional integration (Lam, 2020: 2, 21). Notably, the mid-crisis policy address of 2019 rearticulated two-systems “freedoms” as *concessions*, conditional upon the cessation of contentious politics.

The 2020 address marked a new beginning in more ways than one. In what was widely interpreted as a public humbling, Lam had to postpone her annual address at the 11^th^ hour, after being summoned to Beijing (Cheung, 2020; SCMP, 2020). The rescheduled address took place, not coincidentally, in the wake of a speech from President Xi declaring that the mainland capital of Shenzhen would “take the lead” in the project of regional integration under the Greater Bay Area (GBA) plan. The revised version of Lam’s address, published several weeks later, was notable for its solicitous tone, renarrating 1C2S as a party-state concept. In contrast to portrayals of 1C2S as a kind of shield, *protecting* Hong Kong’s legacy freedoms, and as a *competitive asset* in a world of liberalizing trade and commerce, the 2020 address echoed the party-line, (re)interpretating the compact as an “evolving process” of domestic governance, fortified by a series of categorical imperatives. What had once been a potent symbol of Hong Kong’s late-liberal state of exception was now an instrument for the managed reunification of the city-region with the motherland, all in the service of national development:

[W]e should deeply appreciate the great concept of “One Country, Two Systems” put forth by Mr Deng Xiaoping, which [made] it possible for Hong Kong to **return to the embrace of our motherland**, but also ensured the prosperity and stability of Hong Kong. Last year, the fourth plenary session of the 19th Central Committee of the Communist Party … adopted the decision on “upholding the principle of ‘One Country, Two Systems’, maintaining lasting prosperity and stability of Hong Kong and Macao, and promoting the peaceful reunification of China” as one of the **notable strengths of the state and governance systems of our country** (Lam 2020: 7, emphasis added).

In addition to declarations of fealty to CPC resolutions, Lam went on to reiterate a “corrected” interpretation of 1C2S, as determined by the party’s General Secretary:

President Xi Jinping [has] pointed out that the implementation of “One Country, Two Systems” entailed **an evolving process**, and that Hong Kong had encountered some new circumstances and issues in the process. He mentioned that … it was imperative to have a **correct understanding of the relationship between “One Country” and “Two Systems”** … in accordance with … the Constitution … to always focus on **development as the top priority** [and to] maintain a **harmonious and stable social environment** (Lam, 2020: 1-2, emphasis added).

The new-normal policy addresses of 2020 and 2021 each retain passing references to Hong Kong’s “high degree of autonomy” (Lam, 2020: 6, 8; 2021: 4), but these are preceded and overshadowed by increasingly emphatic statements concerning a “reauthorized” reading of 1C2S. The depiction of 1C2S as “an evolving process” is reiterated in the 2021 address, supplemented to now-routine commitments to “steadfast” and “unwavering” implementation, ostensibly “true to [its] original aspiration” (Lam, 2021: 5). Moreover, in the wake of political upheaval, the geographies of blame and culpability were recast, such that the disorderly state of Hong Kong was tagged to failures of *local* governance, social control, and policing. It had taken “resolute action” on the part of Central Government, “in the face of anti-China, destabilising forces,” to bring Hong Kong “back on track” (Lam, 2021: 5). Nonetheless, Lam felt the need to present a renewed justification for local administration and leadership (however compromised this might appear to be), maintaining that her government remained “in the best position” to secure the correct implementation of policies for Hong Kong (Lam, quoted in Lau, 2021: 1). Transgressing the usually sober register of official policy discourse, Lam repudiated as “nonsense and groundless” the “accusation” that 1C2S principles had been undermined by the imposition—without consultation—of the NSL, along with a new package of restrictive electoral reforms, even as the same text described these as “initiatives of the *Central* Government” (Lam, 2021: 5, emphasis added).

The intemperate and defensive tenor of some of these remarks stand in sharp contrast to the idealistic, first-person statements of governing principles that occupied a prominent place in Lam’s early policy addresses. Whatever relevance that the personal beliefs of the chief executive may once have had, this evidently no longer holds, Lam ceasing even to articulate them. In Xi’s China, there is no space for secondary centers of authority or local interpretations of the party line. The (previously proclaimed) “beliefs” of Hong Kong’s chief executive, like those of provincial leaders and local administrators elsewhere China, were now beside the point.

### Relocating economy: From freedom to integration

Ideologically synonymous with the idea of small-state capitalism, Hong Kong was Milton Friedman’s favorite economy for a reason. Like the colonial administration before it, the HKSAR government continued to make the most of the questionable moniker “freest economy in the world,” bestowed by neoliberal think tanks and their allies (Peck, 2021; Zeng et al., 2021).^9^ With its penchant for liberalized rules of trade, pro-business regulation, low taxes, and limited social-welfare provision, the HKSAR never hesitated to recycle this quintessentially neoliberal narrative, with Lam herself publicly meeting the delegation of “experts” from the Heritage Foundation in what became an annual reaffirmation of Hong Kong’s first-place ranking on the so-called Index of Economic Freedom. For years, the policy addresses predictably hewed to this free-market line. References to the Heritage Foundation and Fraser Institute rankings featured prominently in Lam’s “My Belief” statements of 2017 and 2018, before disappearing without trace. Early in her administration, this limited-government position had been the one from which Lam responded to rhetorical questions concerning the social cost of neoliberal policies,^10^ rejecting any suggestion that her administration might stray from the path approved by the free-market think tanks. Since the 1960s, Hong Kong’s self-declared model of “positive noninterventionism” had been predicated on an *active* posture of restraint, surveillance, and principled stewardship, never equating simply to a do-nothing policy of laissez-faire. Initially, Lam did not demur from this stance:

Some may ask whether the Government’s proactiveness will deviate from the market economy upheld by Hong Kong. My answer is “no”; but a city’s competitiveness is like a boat sailing against the current and it **must forge ahead in order not to be driven back** (Lam, 2018: 5, emphasis added).

True to this defense, Lam’s tentative embrace of proactive governance was an initially circumscribed one, framed largely as a response to a more challenging *external* environment (marked by US-China frictions, growing unilateralism, and destabilized conditions of trade), against which “Hong Kong [could] not stay immune,” even while holding to its limited-government principles (Lam, 2018: 77).

Rather than turbulence in the international system, however, it was a mutiny at home that almost upended Hong Kong’s ship of state: the protests that began in June of 2019 continued to escalate for months. Later that year, Lam’s policy address portrayed the escalating protests as (no more than) “violent incidents [that] have seriously damaged Hong Kong’s international image and undermined its attractiveness to overseas investors” (Lam, 2019: 51). At this point, the default plan seemed to be to revert, as soon as possible, to the status quo *ante* in economic policymaking. This business-as-usual approach—in Lam’s words, “rid[ing] out this storm and “mov[ing] on,” while “maintaining a free market economy”—would involve the enrolment of chambers of commerce and other business organizations in a promotional makeover, thereby “restor[ing] confidence” (Lam, 2019: 25, 51). The storm, however, was not about to blow over, and the best efforts of the local chambers would not suffice. Early in 2020, after the territory had slipped into recession for the first time in a decade, compounded by multiple downgrades of its credit rating, Hong Kong lost its cherished at the top of Heritage’s Economic Freedom rankings, which it had held in an unbroken quarter-century run since the inauguration of the list. The Washington think tank bluntly declared that the economic policy of the territory was now being determined, for all intents and purposes, by Beijing (Heritage Foundation, 2021; Lung, 2021). The HKSAR rejected this “superficial” reasoning, alleging the influence of unspecified “political considerations,” but was forced to face the ironic consequences of having lived, for decades, by this dubious sword (HKSAR, 2021: 20).^11^

After 2019, neither the Heritage rankings nor once-sacred phrases like “free market economy” and “free market principles” would appear in Lam’s policy addresses again. Post-crisis, the spatial horizons of economic-policy discourse pivot from declarations of Hong Kong’s favorable position within global commerce, at the frontier of China’s historic process of opening up, toward the overriding objectives of regional integration and national development. The once confident, cosmopolitan voice Hong Kong, as the self-styled capital of free-market entrepôt economics, has been muted, as the city-state assumes the more subservient role of “a ‘participant’ in domestic circulation and a ‘facilitator’ in international circulation,” under the rule of a “new development pattern … which takes the domestic market as the mainstay” (Lam, 2020: 22). Departing from those spatial imaginaries on which Hong Kong’s reputation was made—in the global vanguard of laissez-faire, free trade, and small-state policymaking, symbolizing an increasingly liberalized future—the new imperative is to serve and support national development. This entails a more “active” role in the project of regional integration under the GBA, leveraging its common-law system in the internationalization of the renminbi, underwriting the tech economy, and facilitating the mobility of talents (K Chan, 2023a; Wu and Wong, 2023).

Hong Kong’s economy, in this governmentalized sphere, has been discursively domesticated. In the process, the “market”, shorn of its adjective “free,” has become an instrumental means of national development, rather than a teleological end. The NSL and “improvements” to the local electoral system are to be credited with “restor[ing] safety and stability in society,” permitting “a new start for economic development” in alignment with the “second centenary goals” of the CPC (Lam, 2021: 24, 79). Meanwhile, the HKSAR has become a different kind of small state, its self-effacingly labelled “current-term Government” deferring simultaneously to the authority of Beijing and the rule of markets.

Driven by changes to the external environment, **guided by the direction of the Mainland policies and led by the market forces**, all these restructuring processes have been attributed to the remarkable acumen, brilliant versatility and “can-do” spirit of Hong Kong entrepreneurs. The current-term Government stresses that we should play the role of a “facilitator” and a “promoter” (Lam, 2021: 24, emphasis added).

In Lam’s final address it would remain necessary, however, to name (and to target) a potential threat to this profitable configuration of driving policies and can-do spirit, those “local political forces against the Central Government [that] have more often than not stood in our way, preventing us from reaping fully the benefits of our country’s development” (Lam, 2021: 24).

**Figure 3.**
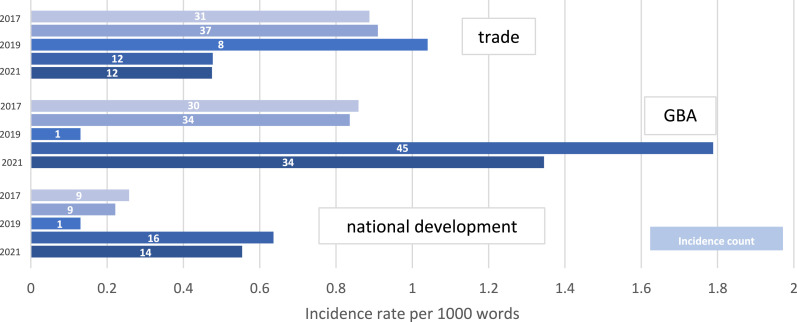
Economic-development keywords: international trade, national development, and regional integration in Hong Kong policy addresses, 2017-2021.

Standardized frequency counts of economic-development keywords, conducted across the five policy addresses and summarized at Figure 3, reveal a decline in references to international trade, with Hong Kong no longer presenting as a standard-bearer for liberalization on the multilateral stage. In fact, the HKSAR has become more likely to protest “foreign interference” (Lam, 2020: 1, 9, 12, 41; Lam, 2021: 79; HKSAR, 2021: 16–17, 22, 54). On the other hand, the dominant motif in economic-development narratives has become that of integration, and the “opportunities” for Hong Kong to capitalize upon (by complying with) China’s “new development pattern.” This is reflected in the references that are made to the encompassing remit of the GBA, positioned as “an entry point” to mainland markets (Lam, 2020: 22).

### Reordering the social: From common values to corrective education

Prior to the crisis of 2019, questions of social welfare and social-policy reform were subordinated to the reigning paradigm of “free market” governance. At the time, building “a harmonious society” was understood to require little beyond a liberal model of economic development, supplemented at the margin by the supportive work of subjects in government, the business sector, and the local community, in “tripartite co-operation” (Lam, 2018: 58). These efforts were accompanied by reassurances that any increase in social spending would be managed according to the principles of fiscal restraint, which in Lam’s second policy address took the form of call-and-response exchange in classically neoliberal terms: “Some may … question whether the Government [is] embarking on a road to a welfare society? My answer is ‘no’” (Lam, 2018: 5).

During her 2017 campaign and initial period in office, Lam prioritized education, including the embrace of “values education,” with the goal of nurturing “future generations into quality citizens who are socially responsible and equipped with a sense of our national identity, a love for Hong Kong and an international perspective” (Lam, 2017: 40). This must not extend, she insisted, the politicization of education, since the sector should properly remain “professional-led,” with the government and the community each respecting the boundaries of teachers and education professionals. Imploring that “we should treat our teachers nicely,” Lam declared that the social tensions around schooling were now safely things of the past, such that “education is re-emerging as education” (Lam, 2017: 2; Lam, 2018: 50).

The issue of education policy disappeared in the 2019 address, which was restricted to matters of social order and (acknowledged) grievances relating to the cost and availability of housing, “the toughest livelihood issue facing Hong Kong society” (Lam, 2019: 5). The wider raft of concerns that had brought protesters onto the streets were pointedly not addressed, the administration’s gratitude instead being reserved for police officers, transportation and maintenance workers, and those ordinary citizens who had remained home and off the streets. On the other hand, the concerns of Hongkongers that had participated in demonstrations, marches, and rallies, joining what was in effect a broadly-based and heterogenous, if leaderless, social movement, were not only overlooked; they were elided with—*and reduced to*—the malign intentions of a violently-inclined minority, whose headline-grabbing actions overshadowed the effective articulation of a wider social claim.

A handful of rioters initiated attacks and sabotages in an organised and planned manner. They doxed and beat people holding different views, spreading chaos and fear in Hong Kong (Lam, 2019: 1)

In the midst of the crisis, Lam emphatically posed the questions, “[W]ill Hong Kong return to normal? Is Hong Kong still a place we can live in peace?”

**Figure 4.**
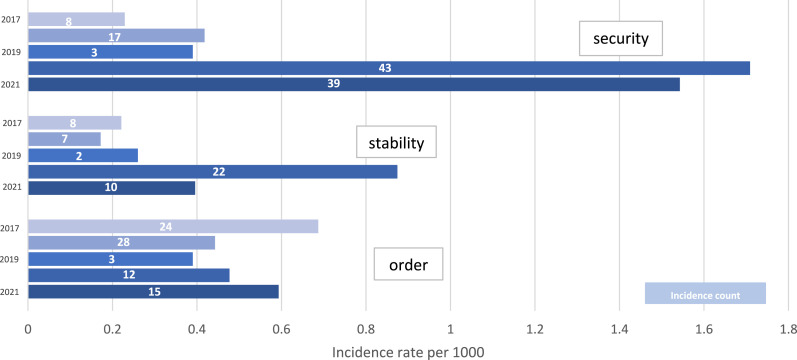
Social order keywords: security, stability, and order in Hong Kong policy addresses, 2017-2021.

In the post-2019 addresses, “stability” and “security” became watchwords, the connotations of “order” being correspondingly reworked in illiberal terms (see Figure 4). What the protests had exposed, according to this interpretation, have nothing to do with widespread misgivings about Beijing’s overreaches into the civic life of Hong Kong, but instead represent “an obvious gaping hole in national security [with] significant risks to the country” (Lam, 2020: 9). This is Lam’s justification for Beijing’s decisive intervention, which is named and affirmed prior to a depiction of a “return to order” that is curiously actorless, the ending of chaos and ridding of disruptive elements being portrayed in passive and intransitive terms:

**[T]he Central Government has no alternative but to step in** and take action … [The NSL] has been remarkably effective in restoring stability in Hong Kong: advocacies of “Hong Kong independence” and collusions with external forces have progressively subsided; some of the prominent figures have kept a low profile; radical organisations have ceased operation or dissolved; those who are suspected of violating the law have absconded; and street violence is significantly on the decline (Lam, 2020: 9–10).

Lam’s final address once again credited the “resolute action” of Central Government, reiterating the mantra that “chaos has ended and social order has been restored” (Lam, 2021: 13). Among the remaining (substantial) duties confronting the HKSAR Government, however, was the imperative of “strengthening national security education and raising law-abiding and national security awareness of Hong Kong people, in particular the youth” (Lam, 2021: 7).

Lam’s post-crisis policy addresses recenter education as a space of active intervention, defined as a project of restoration in the face of the radicalized condition of “a minority of our students” (Lam, 2021: 68). Presuming to speak on behalf of the citizens of Hong Kong, Lam declares that, “We cannot bear to see that with the infiltration of politics into school campuses, students are drawn into political turbulence or even misled to engage in illegal and violent acts,” which is read in this context to imply that “the law-abiding awareness of some young people is weak and that positive values such as mutual understanding and mutual respect are lacking” (Lam, 2020: 73). In the process, “restoring Hong Kong’s educational order” is reframed as a national-security priority (Lam, 2021: 68). Reversing her earlier insistence on depoliticization, Lam (2020: 72–73) “stress[ed] that the role of the Government in education is not merely a provider of resources, but also a policymaker, administrator, and regulator.”

The revamped mission for Hong Kong’s education service, from this perspective, is simultaneously economizing (“building up a talent pool,” encouraging mobility across GBA labor markets) and moralizing (“building a good character,” and cultivating “moral virtues”). In the 2020 address, Lam (2020: 73) declared that “moral development is … the foundation of education. In fact, deepening students’ understanding of the history, culture and developments of our country, and strengthening education on the Constitution and the Basic Law are the fundamentals for fostering their sense of national identity and awareness of national security.” Starting at an early age, there is (now) a pressing need to inculcate the “*correct* understanding of the history and culture of our country” (Lam, 2021: 71, emphasis added). A new code of conduct for teachers would be introduced, emphasizing patriotic education.^12^ In her final address, Lam (2021: 8) announced the removal of “Liberal Studies” from the school curriculum, to be replaced by a new subject, “Citizenship and Social Development,” centered on the theme of “Hong Kong under ‘One Country, Two Systems’.” The chief executive reported that she had personally visited schools to observe these newly authorized lessons, even making herself personally available to lead classes on the constitutional status, powers and functions of the HKSAR under the Basic Law (Lam, 2021: 8).

## Conclusion: special administration at the limit

Carrie Lam’s turbulent and transformative term as Hong Kong’s chief executive will likely be remembered as one that redefined the HKSAR’s quasi-autonomous mode of government. This unique model of special administration was never truly equal to the often-contradictory task of serving “two masters,” and may have never been more than one metastasizing crisis away from local-state failure (Lui, 2020; Vines, 2021). The first half of the 50-year span projected by the 1C2S agreement was characterized by an atmosphere of late-neoliberal stasis—with an executive-led special administration operating under truncated (if not real-time) horizons, managing with leftover technologies of rule, the legacy of a colonial model of liberal administration, towards a future neither fully knowable nor apparently nameable. The second half of the 1C2S mandate, now just beginning, will inevitably trace its indecorous origins to a crisis more suppressed than resolved, and to an historic pivot into a securitized scheme for the “patriotic” governance of Hong Kong, under the close supervision of the party-state, one that seems destined to remain inhibited by deficits in local political capacity and social legitimacy.

Although the 1C2S formula retains its official status—increasingly subject to performative deference, albeit in “corrected” form—a distinct transformation is nevertheless evident, from that extended period in which the Basic Law recycled the ambiguous promise of “borrowed time,” truncating and postponing questions about Hong Kong’s future, to a radically new normal under which the principles of “one-country” integration and securitized, party-state rule are paramount. Exercising its constitutional authority through the provisions of the Basic Law, Beijing has assumed ownership of this future, bearing the responsibility for managing Hong Kong’s contradictory incorporation, between a top-heavy model of financialized capitalism on the one hand and a restructured version of the socialist market economy on the other. Meanwhile the official voice and authorized actions of the HKSAR government are increasingly synchronized with those of the party-state. This is not to say that the two are entirely coterminous, or that there is no longer a “filter” between one-country interests and those of Hong Kong. Translation, adaptation, and mediation continue to occur, but the filter has been recalibrated so as to prioritize performances of patriotic governance and projects of developmental integration, now that “the mainland’s way of interpreting and enforcing the Basic Law will hold sway for years to come” (Vukovich, 2022: 4). In this context, the spaces of quasi-autonomous governance that were so dramatically curtailed during Carrie Lam’s term of office are being subjected to further constriction and control, in a manner as much self-imposed as super-imposed, in the loyal administration of John Lee.

When Carrie Lam presented what was to be the last of her policy addresses, in October 2021, she expressed confidence in Hong Kong’s future. Having steered the territory through an unprecedented crisis, testing the two-systems regime to its limit, the future could now be reclaimed:

[C]haos has ended and social order has been restored. We are now embracing a new era where we can focus on economic development. The HKSAR Government should be visionary and resolute in mapping out the future of Hong Kong in a proactive manner (Lam, 2021: 13).

Hong Kong’s new future, even if it is not what it used to be, is however hardly any easier to map. Having spent decades in pursuit of the journey without end that is free-market governance, the territory would now seem to be trained on a different destination, one defined by the overriding project of “national rejuvenation” and the “second-century” ambitions of the party-state. For many observers, Carrie Lam’s legacy will forever be synonymous with the mismanagement of disorder. It may also be remembered as a pivotal moment: a crisis-mediated transition from one order to another.

## Notes

1. According to the Global Press Freedom Index (https://rsf.org/en/index), Hong Kong has plummeted from a top-20 position in the world in 2002, when its media ecosystem was the most liberal in Asia, to a 2023 ranking of 140.

2. From the rich literature on protest discourses in Hong Kong, see Flowerdew (2012); Flowerdew and Jones (2016); Ng (2020); Bhatia and Ross (2022); Ho and Chiu (2022).

3. With increased insistency, Hong Kong is now exhorted—including by senior members of the Politburo Standing Committee of the CPC—to “strengthen patriotic education at home,” while endeavoring to “tell Hong Kong stories well” abroad, including the “countering [of] Western accusations” and the assertion of China’s geopolitical interests (Wong and Wu, 2023: 1; K Chan, 2023a: 4). In the words of Zhao Leji, the third-ranking official of the Politburo, Hong Kong must “strive to unite all forces to pursue national development,” proactively demonstrating a willingness to make “new contributions” (quoted in Wu and Wong, 2023: 1).

4. On the Umbrella movement, see Flowerdew (2017) and Lee and Sing (2019).

5. The five demands were: full withdrawal of the extradition bill; retraction of the characterization of protests as riots; release and exoneration of arrested protesters; establishment of an independent inquiry into police behavior; and the resignation of Carrie Lam, followed by the introduction of universal suffrage for the Legislative Council and the chief executive elections.

6. Coverage in the China Daily (2019: 8), an extension of the CPC’S propaganda department, was indulgent: “It is not an easy job to release a Policy Address in a tough time … [Lam] got the task done by making the Policy Address concise and focused.”

7. See Fong (2022), Fung and Wong (2023). In July 2002, President Trump issued Executive Order 13936, imposing sanctions on Carrie Lam and 10 other officials (mainly from the HKSAR, including the future chief executive, John Lee, then head of security, along with members of the Liaison Office and the State Council’s office for Hong Kong), for “undermining Hong Kong’s autonomy and restricting the freedom of expression or assembly.”

8. Hong Kong is the only part of China that the CPC formally recognizes as, indeed declares to be, “capitalist” (see Xi, 2022: 50).

9. In circular fashion, the indices developed by the Heritage Foundation (with the *Wall Street Journal*) and the Fraser Institute (with Milton Friedman) were both literally and metaphorically indexed to an idealized reading of Hong Kong’s free-market culture, fiscal conservativism, and stunted welfare state.

10. Adopting the identity of a roving advocate for the free-market cause, Lam stated: “When attending different international conferences in the past year, I emphasised time and again that the practice of capitalism and market economy in Hong Kong was not in conflict with the Government’s social policies to actively improve people’s livelihood” (Lam, 2018: 58).

11. This same report, having denounced the Heritage rankings, went on to boast of Hong Kong’s elevated position in *other* rankings, measuring not only economic competitiveness, but also public safety and “respect for law and order” (HKSAR, 2021: 45).

12. Introduced in 2022, the new code requires teachers to promote national education, while reporting potentially illegal activities or “morally deviant information” (Ma and Yiu, 2022: 5). An independent regulatory body, the Council on Professional Conduct in Education, was also disbanded, for alleged failures to act on cases relating to the period of unrest.
